# Cryptically Patterned Moths Perceive Bark Structure When Choosing Body Orientations That Match Wing Color Pattern to the Bark Pattern

**DOI:** 10.1371/journal.pone.0078117

**Published:** 2013-10-24

**Authors:** Chang-ku Kang, Jong-yeol Moon, Sang-im Lee, Piotr G. Jablonski

**Affiliations:** 1 School of Biological Sciences, Seoul National University, Seoul, Republic of Korea; 2 Institute of Advanced Machinery and Design, Seoul National University, Seoul, Republic of Korea; 3 Centre for Ecological Research (previous Institute of Ecology), Polish Academy of Sciences, Dziekanow Lesny, Poland; University of Arizona, United States of America

## Abstract

Many moths have wing patterns that resemble bark of trees on which they rest. The wing patterns help moths to become camouflaged and to avoid predation because the moths are able to assume specific body orientations that produce a very good match between the pattern on the bark and the pattern on the wings. Furthermore, after landing on a bark moths are able to perceive stimuli that correlate with their crypticity and are able to re-position their bodies to new more cryptic locations and body orientations. However, the proximate mechanisms, i.e. how a moth finds an appropriate resting position and orientation, are poorly studied. Here, we used a geometrid moth *Jankowskia fuscaria* to examine i) whether a choice of resting orientation by moths depends on the properties of natural background, and ii) what sensory cues moths use. We studied moths’ behavior on natural (a tree log) and artificial backgrounds, each of which was designed to mimic one of the hypothetical cues that moths may perceive on a tree trunk (visual pattern, directional furrow structure, and curvature). We found that moths mainly used structural cues from the background when choosing their resting position and orientation. Our findings highlight the possibility that moths use information from one type of sensory modality (structure of furrows is probably detected through tactile channel) to achieve crypticity in another sensory modality (visual). This study extends our knowledge of how behavior, sensory systems and morphology of animals interact to produce crypsis.

## Introduction

Many prey animals have cryptic patterns that resemble background and reduce detection by predators [1-3]. Camouflage can be achieved through a range of mechanisms including background matching, disruptive patterns or countershading [3,4]. Many studies focus on the effectiveness of the morphological characteristics, colors or patterns in providing crypticity [5-8], but behavioral mechanisms contributing to crypsis are less studied. In many cryptic prey animals, the color/pattern of animal body is fixed and the behavior of animals (e.g. choice of resting position or orientation) finally determines the degree of match between the animal body and the background (e.g. moths [9]). Therefore it is important to investigate behavior of animals to fully understand the crypsis of animals in natural environment.

Among many cryptic animals, moths have been extensively studied in terms of behavioral preferences for the background substrate. Most of the studies of moth preferences have explored two types of behavior: i) selection of a matching background in terms of color [10-14] or ii) selection of an appropriate resting orientation [9,12,15-17] with regard to the orientation of the background pattern. These previous studies suggest that moths prefer to rest on the background of similar color to their own. This preference for certain color does not seem to result from direct comparison between their wing color/pattern and the current background, but rather seems to be a pre-determined (e.g. genetically) trait shaped by natural selection [18,19]. It has also been shown that some moths orient their bodies adaptively to maximize their crypticity by matching the pattern on their wings to that of the background [9,12,15-17]. 

On the other hand, we are aware of only one study of proximate behavioral mechanisms that result in such a match. Sargent [20] presented experimental visual and tactile stimuli to see how two moth species (one geometrid and one noctuid) respond to simple directional elements of the background. He proposed that tactile cues and background-independent cues are used by the bark-like moths in finding resting positions and body orientations. 

Our recent studies on two geometrid species have shown that these moths are not only able to find a position and a body orientation that provide good camouflage [9], but that they are also more likely to re-position their bodies to a more cryptic spot if their original landing positions do not provide good visual crypticity [21]. Moth’s vision is unlikely to be important here because visual recognition of fine bark pattern might be difficult for the moths due to low resolution of their compound eyes [22]. Additionally, they cannot view their wings and the bark pattern from the view point of the visually hunting predators, which is crucial to determine the level of cryptic match between the wings and the bark. This indicates that they may perceive non-visual stimuli, such like the ones suggested by Sargent [20], that may be correlated with their current degree of visual crypticity at a resting position. We asked whether the non-visual cues proposed by Sargent [20] are sufficient to explain the re-positioning behavior of these species [9,21]. Furthermore, we expanded the Sargent’s classical experimental design by testing the additional hypothesis that moths could use the bark curvature to orient their bodies.

**Figure 1:**
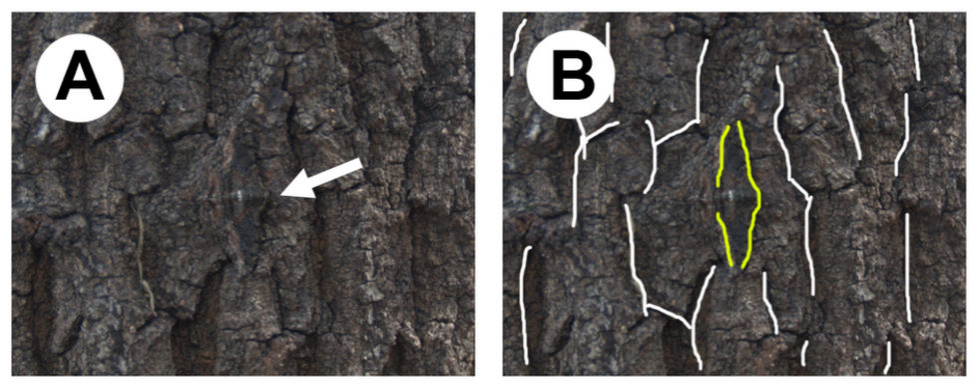
Photographs of a moth on a trunk of oak tree. Both (**A**) and (**B**) show the apparent match between the directions of the furrows in the bark and the patterns on moth’s wings. The arrow in (**A**) indicates the head of the moth. The lines in (**B**) show the direction of the main furrows in the bark (white lines) and the direction of main pattern on the moth (yellow lines).

In this study, we use one of our previously studied geometrid moths, *Jankowskia fuscaria* (Leech 1891), to investigate the cues that these moths use to orient their bodies on a tree trunk. After landing on a tree trunk, *J. fuscaria* performs re-positioning behavior [9]. *J. fuscaria* re-positions its body on the bark by repeatedly lifting and lowering its wings and changes its resting spot and body orientation to reinforce its crypticity. This suggests that *J. fuscaria* may use sensory cues from the background to re-position their bodies to a more cryptic position. The key characteristics of *J. fuscaria* that we utilize in this study are the following: i) the moths actively search for the resting positions and body orientations on the background substrates [9], and ii) they have a tendency to orient themselves towards either of two sides (head pointing towards left or right from the point of view of an observer) on tree trunk after the completion of this re-positioning behavior [9]. If not disturbed during the day, they stay in this position until the following night. The position and body orientation at these chosen sites provide good crypticity because the patterns on the moth’s wings match the main direction of visual patterns on the vertical tree trunk (Figure 1). This active preference for non-random resting orientation suggests that moths may be able to recognize some characteristics of the substrate and use those cues to orient their bodies. We asked what cues are used by *J. fuscaria* during re-postioning behavior, and tested whether *J. fuscaria* uses trunk curvature, visual pattern, or directional three-dimensional structure of crevices or furrows on a trunk.

## Materials and Methods

### Collection of moths and experimental arena

Moths were collected just before dawn (about 4 AM) near the black lights that we setup in the forest (university forest which is preserved for research purpose; no permission is required for members). They were kept individually in small plastic containers with sugar-moistened tissue and used in experiments on the following day. Experiments were conducted under thick forest canopy between 0800 and 1800. To prevent escaping (flying away) of moths during the experiments, we set up a tent made of mosquito net and performed the experiments inside the mosquito net structure. The mosquito net was considerably transparent and visual patterns of a log and visually patterned experimental backgrounds were well visible. The mosquito net only slightly decreased the light intensity without changing the spectral properties of the light (Figure S1), so that we considered the effect of mosquito net on moths’ behavior (choice of a resting spot and body orientation on the backgrounds) negligible. After each experiment, some of the tested moths were frozen for another study and the others were released at a distant location (further than 2 km away) to avoid re-capturing of the same individual. All experiments were performed in Aug-Sep 2011 at Choosan field station, Mt. Baekwoon, South Korea (N35°,01’,54.30”; E127°,36’,22.30”).

### General scheme of experiments

We made several experimental backgrounds, each of which was designed to have a single characteristic that may be used by moths on tree trunks: visual pattern, directional furrow structure, and curvature. The backgrounds were presented in two treatments: vertical treatment which imitates a vertically standing trunk, and horizontal treatment for which we rotated the vertical treatment by 90 degrees. By exploring the resting orientation of moths on each background in both treatments, we asked i) whether moths orient themselves by using the cues from the background tree trunk, and ii) which sensory components of the background moths use to orient. 

Each experiment comprised two treatments; vertical and horizontal. In the vertical treatment, we presented background cues in a natural manner, as they are present on a vertical tree trunk. In the horizontal treatment, we rotated the presentation of the background cues by 90°. For all the experiments, we assumed that i) if moths perceive the background cues provided in the vertical treatment and if they use the given cues to orient their bodies in a manner providing a cryptic match between the wings and the bark pattern, the resting orientation of moths would be the same as the orientation in nature (towards either of two sides), and ii) after we rotate the background, the resting orientation of moths should follow the orientation of background cues if the moths use the given background cues in each experiment (visual pattern, structure, and curvature). Curvature was tested in two conditions: thick trunk (with low curvature) and thin trunk (with high curvature). In each experiment, we measured the angle between the orientation of moth’s head and the vertical line (0° if a moth's head points towards the top; the angle runs in the clockwise direction).

### Experiment 1. Do moths recognize background cues on natural backgrounds?

We first tested whether moths recognize and rely on background cues to position and orient themselves on a natural tree trunk. As the natural substrate, we used a log of an oak *Quercus acutissima*, which is one of the common species at the study site and has prominent directional furrows. The directions of the main furrows were mostly vertical (Figure 1; see Figure S2 for details of the furrow directions). The size of the log was 21 cm (radius) × 67 cm (length) so that it had sufficiently large area for moths to rest on. Two orientations of the log were used as the experimental treatments: vertical and horizontal. For the vertical treatment, we set the log standing upright; for the horizontal treatment, we laid the log down (90° rotated).

We released a moth on the log, and after one hour (one hour is sufficient time for *J. fuscaria* to re-position themselves to final resting spot and orientation [9]) we photographed the moth and captured it. Then, we rotated the log and repeated the same procedure with the same individual. The order of the testing was counterbalanced between moths: half of the moths were tested in the vertical treatment first (in the earlier part of a day) followed by horizontal treatment (in the later part of a day), and the other half were tested in the horizontal treatment first. Hence, the effect of testing time on moths’ behaviors, if present, should not bias the results. From the photographs, we measured the head orientations of the moths. Although most of the moths stayed put once they settled down on the log, few moths flew away during the one hour of waiting time. This resulted in a slight difference in sample sizes between treatment groups (34 moths were tested in both treatments; one additional moth was tested in the vertical treatment).

For testing, it was essential to make the moth land on the background we provided. However it was difficult to let the moth fly to the log willingly and make it land on the background prepared for the experimental test. Therefore, we let the moths walk from the container box onto the log. Although this situation is not natural (usually, moths fly and land on a substrate), the moths sought new resting spots and orientations during walking on the log in a manner similar to normal re-positioning behavior [9]. In order to validate this method of moth release, we compared the orientations of moths on the vertically standing log after released by this method with the orientations of moths on natural tree trunks at the study site after releasing them and letting them fly to the trunks (data collected in 2010 summer). If moths released in our experiments seek out resting orientations in a similar manner as they do in nature, their orientations in the vertical treatment should be similar to their orientations on natural tree trunks. The orientations of moths in the vertical treatment did not differ from the orientations of moths on the natural tree trunks in the forest (Watson test, U^2^=0.05, N_*1*_=29, N_*2*_=35, *P* >0.1, Figure S3). This demonstrates that our experimental procedure of letting a moth walk out of the box onto the substrate resulted in orientations similar to the orientations of moths in a more natural situation whereas a moth flies towards and lands on the substrate. Therefore we decided that our procedure is acceptable for exploring the resting orientation of moths. 

### Experiment 2. Do moths use visual pattern of the background?

**Figure 2:**
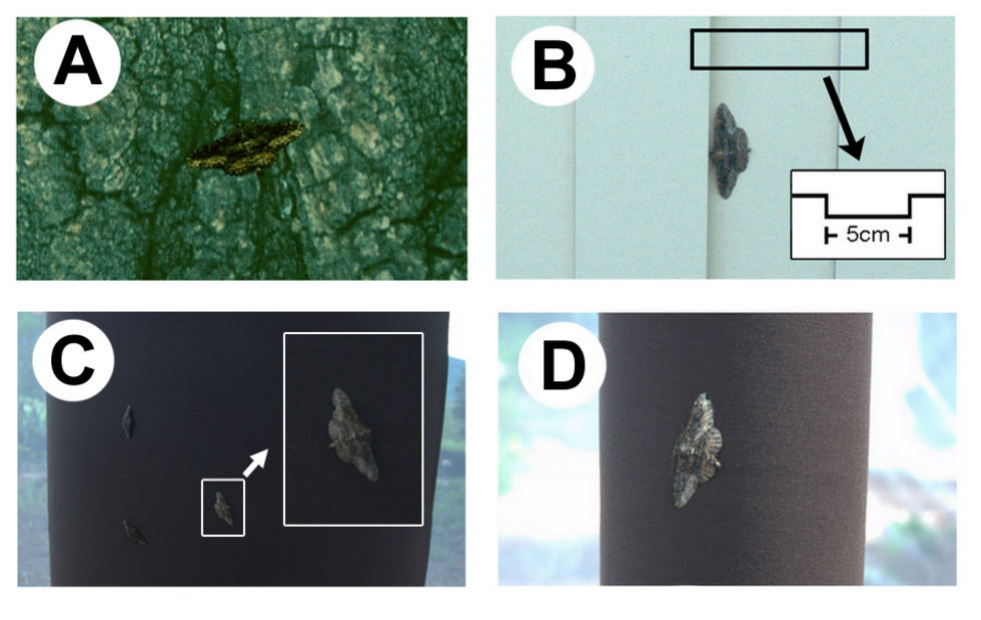
Photographs of moths on the backgrounds that were used in the experiments. These are examples of the moths on the log used in the experiment 1 (A), the printed photograph used in experiment 2 (A), the artificial flat background with with directional structure used in experiment 3 (B), the thin round (high curvature) background used in experiment 4 (C), and the thick round (low curvature) background used in experiment 4 (D). The inset in (**C**) shows a schematic cross-section of the background structure, and the inset in (**D**) shows the magnification of the moth.

To test whether moths solely use visual patterns of tree trunk to position and orient their bodies, we used a printed photo of the bark patterns of the log (the same log that was used in the experiment 1) in real size (YUPO synthetic paper, printed by Mimaki JV4-160 printer). The printed photo was carefully glued on a 66 × 98 cm flat cardboard (see Figure 2A). This background presents the pattern of the tree trunk, but does not have furrow structure or roundness. At a close examination of the printout, we found no directional microstructures or grooves made from paper manufacturing process, printing or gluing, which could affect moths’ behaviors. Reflectance measurements of the real bark and the printout did not show much difference between these two types of background (see Figure S4 for the reflectance data). Although we cannot exclude the possibility that the slight color difference between the printout and the real bark might have affected the choice of a resting site during the experiment (the difference in reflectance affects the background choice of moths [13,15,23]), it is unlikely that it affected the choice of a body orientation of the moths because the distribution of furrow directions, which would affect moths’ orientations, did not differ between the real bark and the printout. The experimental treatments and moth releasing procedures were the same as in the experiment 1. We tested 32 moths in both treatments (each individual in both treatments), and two additional moths in the vertical treatment.

### Experiment 3. Do moths use directional structure of the background?

To test whether moths use the three dimensional directional structure of the tree bark to orient their bodies, we made an array of artificial directional structures that correspond to simplified furrows and crevices of a tree bark. The artificial background consisted of rectangular (98 × 66 cm) flat cardboard paper with either horizontal or vertical narrow strips of cardboard papers (66 × 5 × 0.5 cm) glued parallel to one another with 5 cm spaces between them (Figure 2B). The 5 cm distance between the strips was chosen because it is wider than the wing span of *J. fuscaria* (which is about 3.75 cm for females) and therefore it did not physically restrict the moths from choosing resting orientations nor hinder the moths from walking around. The experimental treatments and moth releasing procedures were the same as the experiment 1. We tested 28 moths in both treatments (each individual in both treatments) and additional three moths in the vertical treatment.

### Experiment 4. Do moths use curvature of the background?

To test whether moths use the curvature of trunk’s outer surface to orient their bodies, we created the backgrounds of high and low curvature. Visual patterns and structural cues were absent in both types of backgrounds. Low curvature mimicked a tree trunk of thick girth (2 m in diameter) and high curvature mimicked a tree trunk of thin girth (40 cm in diameter). These are within the range the circumference of natural tree trunks. Moths are known to rest on both thick and thin trunks [24]. For the low curvature background, we rolled a piece of cardboard paper (66 × 98 cm), made a semi-lunar shape in cross-section, and taped the corners of the paper together to maintain semi-lunar cross-section. Thus the background presented a round surface that is sufficiently broad for the moths to rest on (Figure 2C). For the high curvature background, we rolled a piece of paper (66 cm in length) and fixed its shape using a tape (Figure 2D). For both backgrounds, we covered the surface with brown (bark-colored) linen cloth which had no directional textures. The experimental treatments and moth releasing procedures were the same as the experiment 1. On the low curvature background, 37 moths were tested in both treatments, and additionally 3 moths were tested in the vertical treatment. On the high curvature background, 38 moths were tested in both treatments and additionally one moth was tested in the vertical treatment. 

### Statistical methods

For each of the four experiments, the prediction was straightforward: if the moths recognize the directional cue provided in the experiment, they should orient themselves towards either of two sides in the vertical treatment (like in natural situation, see above for the explanations) and upwards or downwards in the horizontal treatment (90° rotated from vertical treatment). To test this prediction, we compared the distributions of observed moth orientations with the predicted orientations in each treatment. This was done separately for each treatment, which allowed us to use the same individuals for different treatments without committing pseudo-replication at the level of a single statistical analysis (no moths contributed twice to the same statistical analysis) and without tracking the individual ID of moths (we decided to avoid handling the moths to minimize the disturbance by the experimenter). We did not use pairwise comparisons between the horizontal and vertical treatments (which can directly evaluate statistical significance of the observed differences in the orientations between the two treatments) because pairwise comparisons do not evaluate the type of hypothesis we aimed to test: “the moths orient themselves in a specific direction(s)”, and also because this would require individually marking, and disturbing, the moths. Taking all the considerations into account, we think our statistical approach is sufficient for the main purpose of the study, the evaluation of whether moths’ orientations follow the specific orientations predicted for horizontal and vertical treatments separately.

The data was analyzed in several steps. For each experiment, at first, we tested whether the orientations of moths in each treatment are uniformly distributed on a circle (1-360°) using Kuiper’s one sample test of uniformity (nonparametric circular test) to test the null hypothesis that moths do not prefer any orientation. If the result of this test shows that data do not deviate significantly from null distribution, we can conclude that moths do not follow any specific body orientation. If the data did not follow the null uniform distribution, we transformed the original variables (body orientations of moths in each experiment, see below for the basis of the transformation and procedure) and set out to test whether their prevalent body orientations did not differ significantly from the predicted orientation. 

If insects recognized the directional cues provided in the experiments, and if other factors did not bias the moths’ orientations, we expected that the moths in the vertical treatments would orient their bodies horizontally in a bimodal fashion (heads pointed towards right or left; 90 and 270° respectively), and the moths in the horizontal treatments would orient their bodies vertically in a bimodal fashion (head pointed upwards or downwards; 0 and 180° respectively). Hence, for the tests of the hypotheses it did not matter whether a vertically (or horizontally) orienting moths head upward or downward (or leftward or rightward for horizontal treatment) as long as they show a significant tendency to orient their body axes along the vertical (or respectively horizontal) line. Therefore, we transformed the original orientation values to obtain a unimodal distribution. 

The method of transformation followed Zar [25]; the angle was doubled if the doubled angle was less than 360°; if the doubled angle was more than 360° then we subtracted 360° from the doubled angle. By this transformation, both 90 and 270° (towards right or left) were transformed to 180°, and 0 and 180° (upwards or downwards) were transformed to 0°. Then, we performed the Kuiper’s test of uniformity again on the transformed values (null hypothesis: the data are uniformly distributed on a circle). If the hypothesis of uniform distribution was again rejected (thus strongly indicating non-uniform distribution), we used Rayleigh’s test to evaluate whether their body orientations (orientation angle) followed the predictions. Thus, Rayleigh’s test evaluated the null hypothesis of the uniform distribution against the alternative hypothesis that the distribution had a specified mean direction (angle) of 180° (for vertical treatment) or 0° (for horizontal treatment). In the main figure we only show the distribution of the original circular variables but in supplementary material we also show the distribution of the transformed variables (Figure S5).

All these tests were performed for each treatment and each experiment separately so that a single individual contributed a single datum to each analysis. Circular mean (*θ*_*t*_) and circular variance (*V*_*t*_) were calculated from the transformed angles. All the statistical tests were conducted using R 2.15.0, package "circular" (the function "kuiper.test", "rayleigh.test", "watson.two.test"). To analyze distributions of moth orientations between more and less shadowy edges in experiment 3 we used exact binomial test (“binom.test”). 

Some moths were tested in two different types of experiments in order to maintain comparable sample size in each experiment/treatment. However, since we analyzed treatments separately no moths were included twice in the same single statistical test. 

### Ethics statements

This research has been conducted according to relevant national and international guidelines in Seoul National University forest which is preserved for research purpose. The permission for field study is not required for the university members. No protected species were sampled.

## Results

### Experiment 1. Do moths recognize background cues on natural backgrounds?

**Figure 3:**
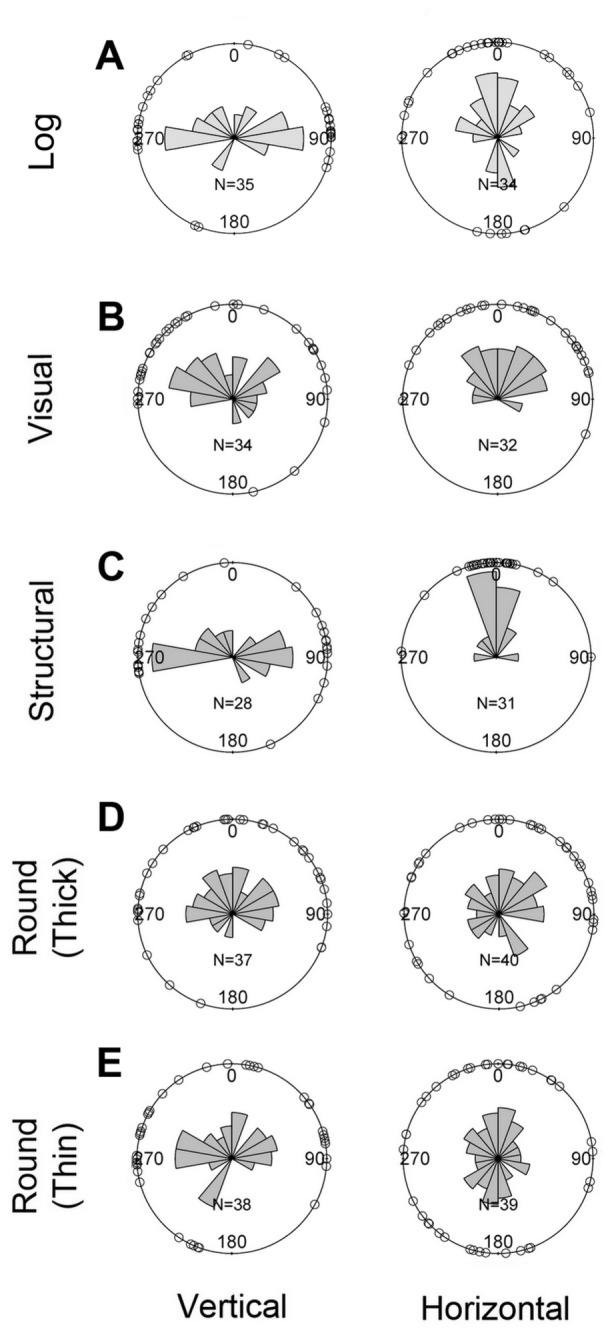
The angular distributions of transformed body orientations of moths in the experiments. The figures show the body orientation of moths on the log (**A**), and the background with visual cues (**B**; experiment 2), structural cues (**C**; experiment 3), low curvature cue (**D**; experiment 4), and high curvature cue (**E**; experiment 4). The rose diagrams (i.e. histogram of directions) show the relative frequency of moth’s head pointing towards the direction of the bar.

In the experiment with the natural log of an oak tree, moths' orientations were not uniform (Kuiper test, vertical, *V*=2.29, *N*=35, *P*<0.01; horizontal, *V*=2.29, *N*=34, *P* <0.01) in a manner that resulted in a match between the direction of the patterning on their wings and the direction of patterning on the bark regardless of the treatment (Figure 1). Their heads were directed towards either side in the vertical treatment (Rayleigh test, *V*=0.58, *N*=35, *θ*_*t*_ =181.49, *V*_*t*_=0.42, *P* <0.001, Figure 3A), or either upwards or downwards in the horizontal treatment (Rayleigh test, *V*=0.47, *N*=34, *θ*_*t*_ =-12.35, *V*_*t*_=0.51, *P*<0.001). 

Additionally, we examined how moths oriented their bodies in relation to the orientation of the nearby furrow (i.e. the head orientation of a moth in relation to the orientation of the closest furrow). Rayleigh’s test with the specified mean direction showed that the moths oriented perpendicularly to the direction of the nearest furrow (see Figure S6 for details). Because furrow orientations follow the orientation of the log (Figure S3), this result is consistent with moths’ heads directed towards the sides in the vertical treatment and upwards or downwards in the horizontal treatment.

These results indicate that moths on the log used some kind of background cues to adjust their orientations according to the orientation of furrows in the bark. 

### Experiment 2. Do moths use visual pattern of the background?

On the visually patterned backgrounds, moths’ orientations had fairly uniform distribution; most angles were distributed in the upper half of the spherical region regardless of the treatment (Figure 3B; Table 1 for statistics). This suggests that the background visual patterns alone may not be crucial in choosing appropriate orientation, and that in the absence of structural cues (such like bark furrows) the moths have a general tendency to orient themselves upwards (Figure 3B).

**Table 1:** The results of the statistical analyses of moths’ orientation.

Type of background	Treatment (sample size)	Kuiper’s one sample test of uniformity on original orientation angles (H_0_: Uniform distribution)		Kuiper’s one sample test of uniformity on transformed orientation angles (H_0_: Uniform distribution)		Rayleigh’s test on transformed orientation angles (H_a_: expected mean orientation angle)	
		*V*	*P*	*V*	*P*	*V*	*P*
Visual	Vertical (34)	2.52	<0.01	1.67	0.05-0.10	NA	
	Horizontal (32)	3.18	<0.01	1.27	>0.15	NA	
**Structural**	**Vertical** (28)	2.01	**<0.01**	2.74	**<0.01**	0.65	**<.001**
	**Horizontal** (31)	4.25	**<0.01**	3.73	**<0.01**	0.75	**<.001**
Round (thick)	Vertical (37)	2.20	<0.01	1.11	>0.15	NA	
	Horizontal (40)	1.70	0.05-0.10	0.98	>0.15	NA	
**Round (thin)**	**Vertical** (38)	1.84	**<0.05**	2.02	**<0.01**	0.28	**0.007**
	Horizontal (39)	1.48	>0.15	1.74	0.05-0.10	NA	

NA indicates ‘Not Analyzed’ because the null hypothesis (H_0_: uniform distribution) was not rejected by the Kuiper’s test. Significant results are marked bold. The significant *P* values in the right-most column (Rayleigh’s test) indicate that moths orient towards either of two sides (left or right) in the vertical treatment, or that they orient towards either upwards or downwards in the horizontal treatment. The original distributions of the moths’ orientations are shown in Figure 3. Bold texts indicate that moths oriented as we predicted (i.e. the moths perceived the cue that we presented; see main text for explanations).

### Experiment 3. Do moths use directional structure of the background?

On the structural backgrounds, moths clearly showed a preference to orient themselves towards either of two sides in the vertical treatment (*θ*_*t*_=184.24, *V*_*t*_=0.35, Figure 3C; Table 1) and to orient upwards in the horizontal treatment (*θ*_*t*_=-5.44, *V*_*t*_=0.25). Many of the moths (50% in the vertical treatment and 74% in the horizontal treatment) positioned themselves similar to the moth in Figure 2B, with their heads and the frontal edge of forewings nearly touching the edge of the experimental structure. We further evaluated the possibility that moths might have responded to the differences between the shadows created by structures. The shadows made by the structure were slightly different between each side (example in Figure 2B), and this might have affected the orientation of moths. To determine if moths paid attention to the shadow differences in the vertical treatment, we categorized the moths as oriented (head direction) towards the more shady or less shady edge. The number of moths that headed towards the more shady edge (8 out of 14) was not statistically different from the moths heading towards less shady edge (6 out of 14; exact binomial test, *P*=0.79), indicating that this visual cue from a slight difference in shadows did not play a major role in moths’ orientation. Additional tests in 2013 showed that moths did not show a preference for artificial vertical structural edges that did or did not create shadows, respectively (see Supporting information text). These results suggest that moths used directional structures to orient their bodies, but that the visually perceived shadows from the directional elements of the structure did not play a role in this behavior.

### Experiment 4. Do moths use curvature of the background?

On the low curvature backgrounds, moths oriented themselves randomly in both treatments (Figure 3D; Table 1). On the high curvature backgrounds, moths showed a tendency to orient towards either of two sides in the vertical treatment (*θ*_*t*_=163.18, *V*_*t*_=0.71, Figure 3E), but oriented themselves randomly in the horizontal treatment (Figure 3E; Table 1).

## Discussion

Our results indicate that moths use cues from the background to adopt the appropriate resting orientation. We have evidence that the details of the substrate’s structure, such like the directionality of crevices and furrows in the bark, influence the resting orientation of moths. Moths tend to orient their heads towards the edge of a crevice/furrow, with the straight-lined frontal wing edges touching (or nearly touching) the wall created by the three-dimensional structures. This results in their body axis orientations roughly perpendicular to the prevalent direction of the furrows, which makes the orientation of wing patterns match the orientation of the bark pattern. The moths in our study had a bias to orient upwards in the absence of structural cues and regardless of the orientation of visual patterns of the background (Figure 3B). In the horizontal treatment of the structural background (Figure 3C), this tendency might have been amplified by the preference to face the edges (horizontal in this treatment). This suggests that the general tendency to orient upwards on the flat backgrounds (in experiment 2 and 3) might be due to factors unrelated to the background structure. We hypothesize that geotaxis or phototaxis may be responsible for the generally upward orientations on flat backgrounds.

One can argue that the single particular log used in the experiments (the properties of the log) might have caused the moths to rest in orientations that differed from the typical orientations in natural situations. However, the resting orientations of the moths on the vertically standing experimental log were very similar to those of the moths resting on natural backgrounds of many different trees (see Figure S3). Additionally, although we used a single log to test all the moths, the furrow structures of the log can be regarded as those of typical oak trees (see Figure S2) because most of the oak trees have similar and predictable furrow structures. Therefore, we do not think that the individual properties of that specific log caused the moths to rest in specific orientations, and we think that the results from the log experiment can be generalized to all oak trunks. One can also argue that, since we continuously used the same backgrounds (one or two backgrounds for experiments) to test all the moths, the initially tested moths might have left some cues (e.g. olfactory) that may have affected the behavior of moths tested later. However even if there was a cue left from the previously tested moth(s), it may affect the choice of the resting spot but not the orientation. Therefore, we think that the orientations of moths were not affected by the repeated use of the same log in the experiments.

What are the sensory mechanisms that moths use to detect the edges and furrows? Perception of background visual patterns to align the patterns on the wings with the patterns on the bark would require the viewpoint of the predators (i.e. from some distances above) rather than that of the moths. Additionally, visual recognition of fine bark pattern might be difficult for the moths because the resolution of their compound eyes is not sufficient to perceive fine-patterns of tree trunk or their wings [22]. Although Sargent [20] suggested that geometrids may use tactile cues to adopt specific resting attitudes, we did not precisely determine how moths perceived the three-dimensional structural elements of the bark in experiment 1 or the artificial structure in experiment 3. It is possible that they used tactile cues, which probably were perceived through antennae [26] (and maybe through the frontal edge of the wings). They did not seem to pay attention to a difference between nearby shadows, and they did not seem to differentiate between the edges that created shadows and those that did not (Text S1). Hence, the results suggest that regardless of visual patterns of the background the three-dimensional structural cues were needed for the moths to choose their orientations that provide camouflage in natural situations. Therefore, we hypothesize that moths detect the structural cues using tactile, rather than visual, sensory channel. 

Our results also suggest that curvature may play some additional role in moth orientation, but only when the curvature is prominent (thin trunks) and presented vertically (probably closer to natural situations). We noted that the distribution of moth orientations on high curvature background appears to be more dispersed (Figure 3E) than that on the background with artificial structures (Figure 3C). This suggests that any effect of curvature is weaker than the effect of directional structure of the background. In natural situations, curvature could be easily disrupted and masked by the existence of furrows especially when a moth is on a thick trunk with prominent furrows. Therefore, we believe that in many natural situations furrow structure is the cue that moths use because it reliably correlates with the visual patterns that match the patterns on moth wings. 

In summary, we showed here that *J. fuscaria*’s behavior leads to the alignment between the direction of wing patterns of the moths and the direction of the furrow structures on natural tree trunks as well as on the artificial experimental backgrounds with directional structures. Many other species of geometrid moths perform similar re-positioning behavior (unpublished data, CKK), and the similarity between our results and those of Sargent [20] indicates that the use of structural cues from the background can be a general mechanism for the bark-like geometrid moths to adopt appropriate resting orientations that provide camouflage. Although it is clear that moths do not perform direct visual comparison between the patterns on their wings and those on the bark, the exact nature of the sensory mechanism responsible for achieving this visual crypticity is not well understood. Hence, our findings open the possibility to study how the moths use information from one type of sensory modality (hypothetically the tactile channel) to achieve crypticity in another sensory modality (visual) and extend our knowledge of how behavior, sensory systems and morphology of animals interact to produce crypsis.

## Supporting Information

Figure S1 **The shape of spectra of natural light and the light through a mosquito net.** This figure shows the relative intensity of light (max intensity = 1) measured by spectrometer (USB2000+, Ocean Optics). The light intensity was measured under forest canopy outside the mosquito net (straight line) and inside of the experimental tent made of the mosquito net (dotted line). The shapes of the light spectrum were similar between natural light and the light through a mosquito net.
(TIFF)

Figure S2 **The directionality of furrows on the log used in the experiment 1.** We randomly selected 50 furrows on the log (**A**). For each furrow we measured the angle between the imaginary line running upward along a furrow and the vertical line (0° if the direction was vertical). Possible values were within the range 0-90 and 270-360 degrees. (**B**) shows the distribution of the furrow orientations. The result shows that the orientations of furrows were closely matching the general orientation of the log.
(TIFF)

Figure S3 **Angular distributions of moth orientations on natural tree trunk and vertically standing log.** (**A**) shows the distribution of moth orientations on tree trunks in natural situation (observed by releasing-following procedure). (**B**) shows the distribution of moth orientations on vertically standing log in the experiment 1. The two distributions did not differ from each other statistically (Watson test, U^2^=0.05, N_*1*_=29, N_*2*_=35, *P* >0.1).
(TIFF)

Figure S4 **The comparison of reflectance between the tree bark and the printed bark pattern.** This figure shows the reflectance spectra of the tree bark (bold line) and the printed photo of the tree bark (used as the visually patterned background in experiment 2; dotted line). We used USB2000+ spectrometer (Ocean Optics), DT-Mini 2 tungsten light source (Ocean Optics), and Labsphere USRS-99-010 standard to measure reflectance of the oak bark and printed pattern.
(TIFF)

Figure S5 **The distributions of transformed body orientations of moths in the experiments.** The figures show the body orientation of moths on the log (**A**), and the background with visual cues (**B**; experiment 2), structural cues (**C**; experiment 3), low curvature cue (**D**; experiment 4), and high curvature cue (**E**; experiment 4).
(TIFF)

Figure S6 **The distribution of head orientations of moths relative to the orientation of the nearest furrow.** These figures show the distribution of head orientations of moths on vertical (**A**), and horizontal (**B**) log. The angle between the head orientation of a moth and orientation of the closest furrow (closest to the moth’s head) was defined as 0° if moth’s body axis runs parallel to the closet furrow, and to 90° if moth’s head pointed perpendicularly towards the furrow. Rayleigh’s test with specified mean direction (towards 90 °) showed that the moths mostly oriented perpendicularly to the direction of the nearest furrow (vertical treatment: *N*=35, *V*=0.91, *P*<0.001; horizontal treatment: *N*=34, *V*=0.82, *P*<0.001).
(TIFF)

Text S1 **Methods and results of the additional tests in experiment 3.**
(DOCX)
