# Supplementary figures and images for: Cryptically Patterned Moths Perceive Bark Structure When Choosing Body Orientations That Match Wing Color Pattern to the Bark Pattern
Source: PLoS One. 2013 Oct 24;8(10):e78117. doi: 10.1371/journal.pone.0078117 (PMC3813426; doi:10.1371/journal.pone.0078117)

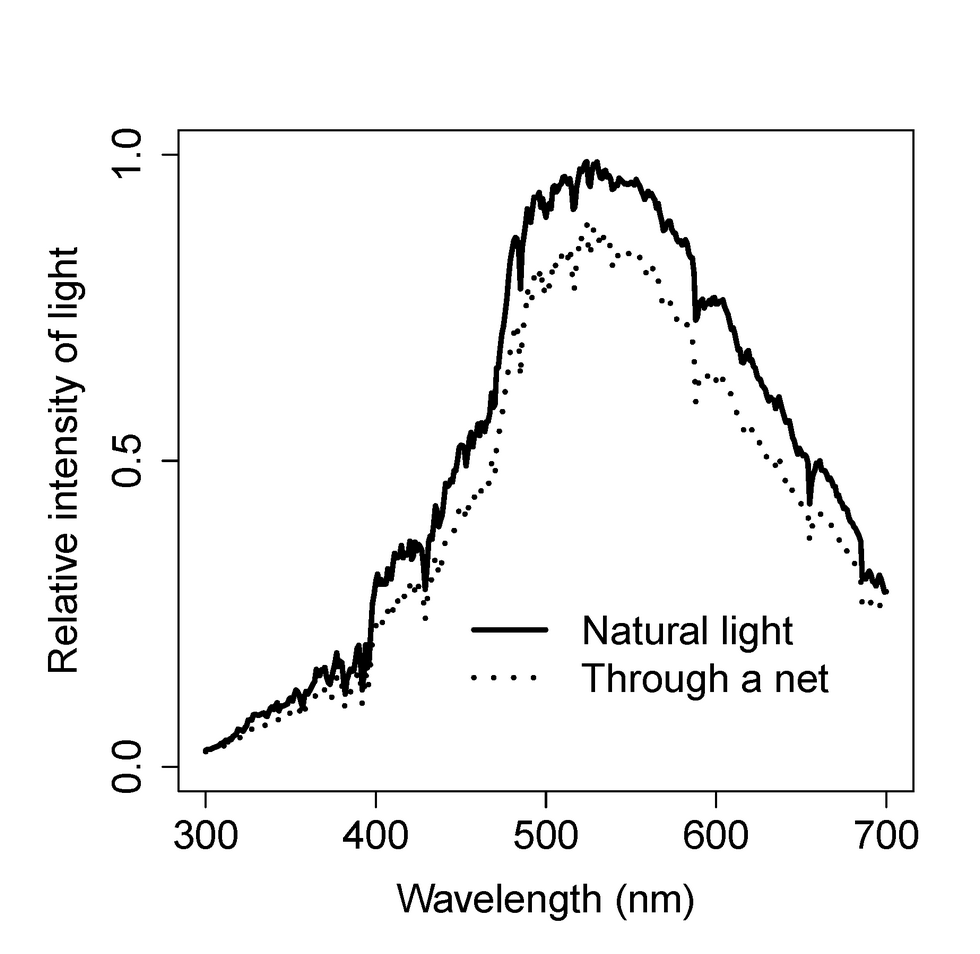

Supplement: Figure S1 — The shape of spectra of natural light and the light through a mosquito net. This figure shows the relative intensity of light (max intensity = 1) measured by spectrometer (USB2000+, Ocean Optics). The light intensity was measured under forest canopy outside the mosquito net (straight line) and inside of the experimental tent made of the mosquito net (dotted line). The shapes of the light spectrum were similar between natural light and the light through a mosquito net. (TIFF) [file pone.0078117.s001.tiff]

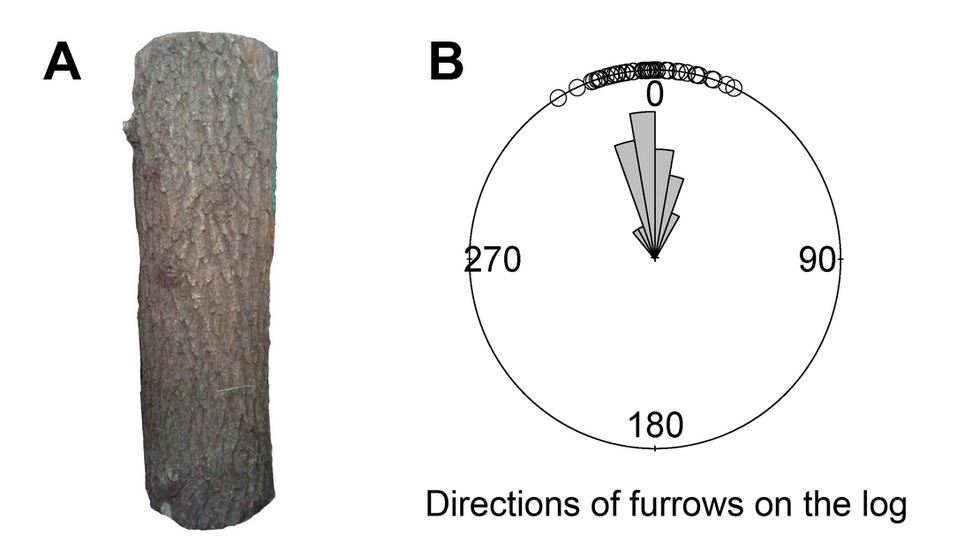

Supplement: Figure S2 — The directionality of furrows on the log used in the experiment 1. We randomly selected 50 furrows on the log (A). For each furrow we measured the angle between the imaginary line running upward along a furrow and the vertical line (0° if the direction was vertical). Possible values were within the range 0-90 and 270-360 degrees. (B) shows the distribution of the furrow orientations. The result shows that the orientations of furrows were closely matching the general orientation of the log. (TIFF) [file pone.0078117.s002.tiff]

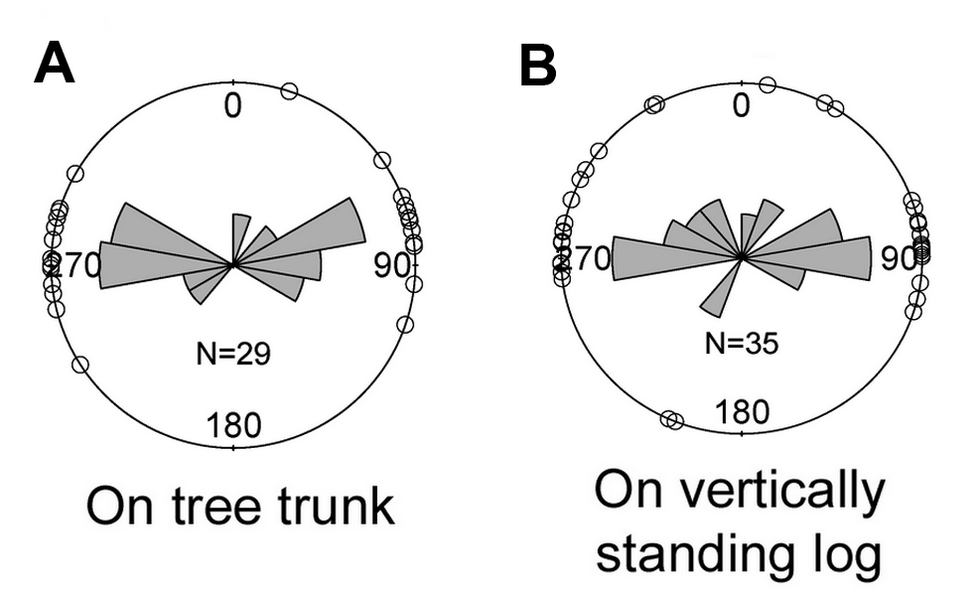

Supplement: Figure S3 — Angular distributions of moth orientations on natural tree trunk and vertically standing log. (A) shows the distribution of moth orientations on tree trunks in natural situation (observed by releasing-following procedure). (B) shows the distribution of moth orientations on vertically standing log in the experiment 1. The two distributions did not differ from each other statistically (Watson test, U2=0.05, N1=29, N2=35, P >0.1). (TIFF) [file pone.0078117.s003.tiff]

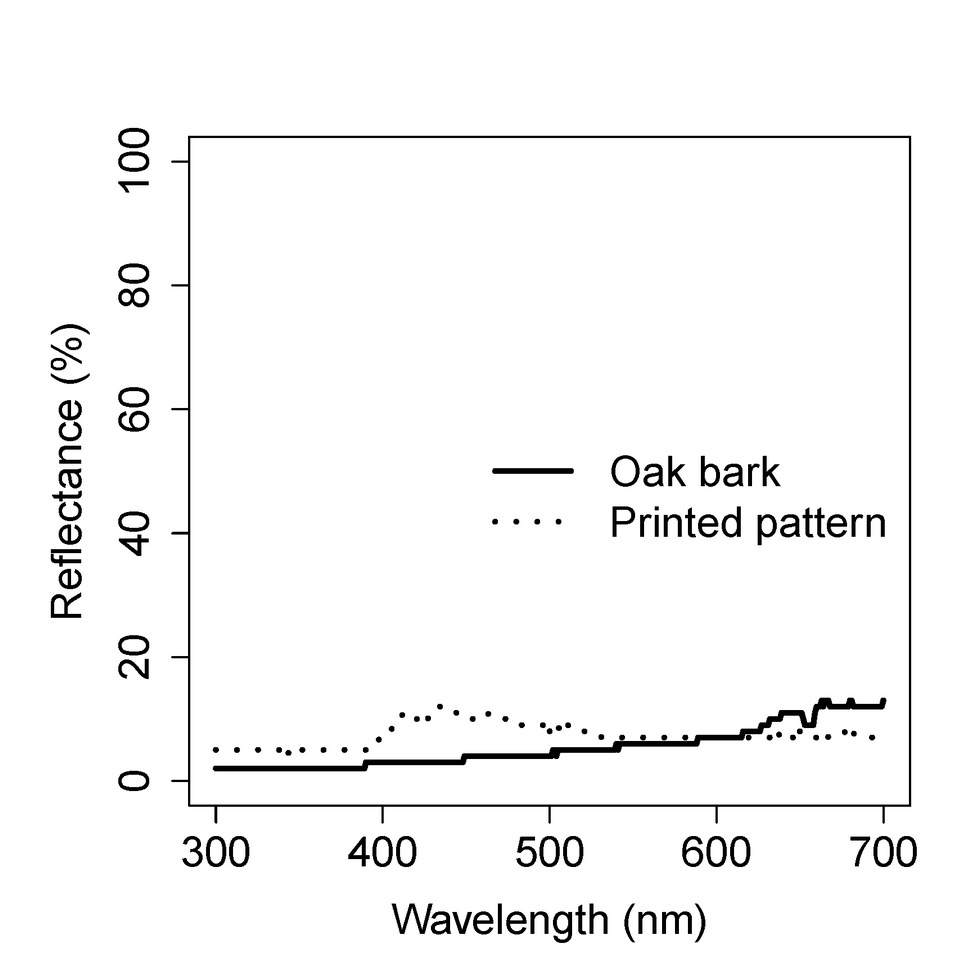

Supplement: Figure S4 — The comparison of reflectance between the tree bark and the printed bark pattern. This figure shows the reflectance spectra of the tree bark (bold line) and the printed photo of the tree bark (used as the visually patterned background in experiment 2; dotted line). We used USB2000+ spectrometer (Ocean Optics), DT-Mini 2 tungsten light source (Ocean Optics), and Labsphere USRS-99-010 standard to measure reflectance of the oak bark and printed pattern. (TIFF) [file pone.0078117.s004.tiff]

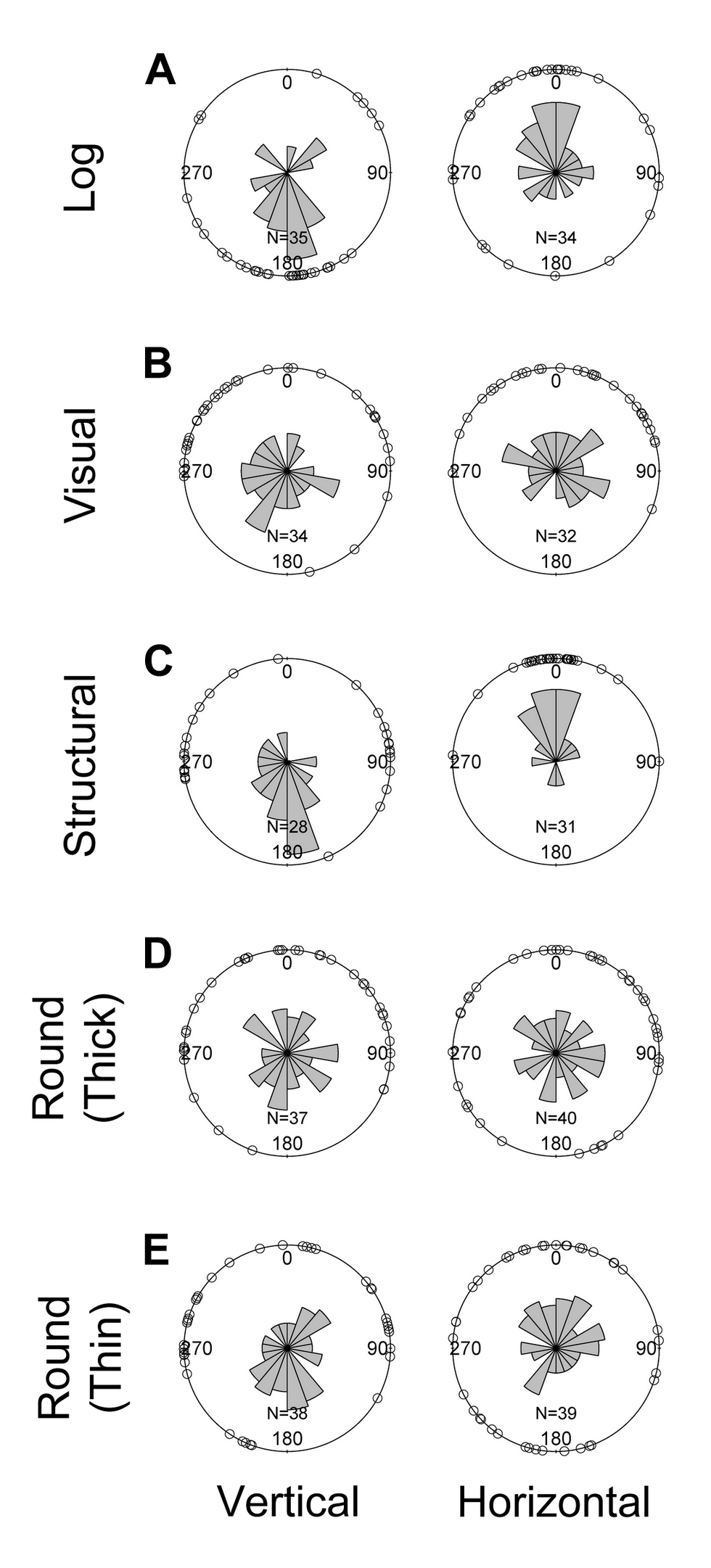

Supplement: Figure S5 — The distributions of transformed body orientations of moths in the experiments. The figures show the body orientation of moths on the log (A), and the background with visual cues (B; experiment 2), structural cues (C; experiment 3), low curvature cue (D; experiment 4), and high curvature cue (E; experiment 4). (TIFF) [file pone.0078117.s005.tiff]

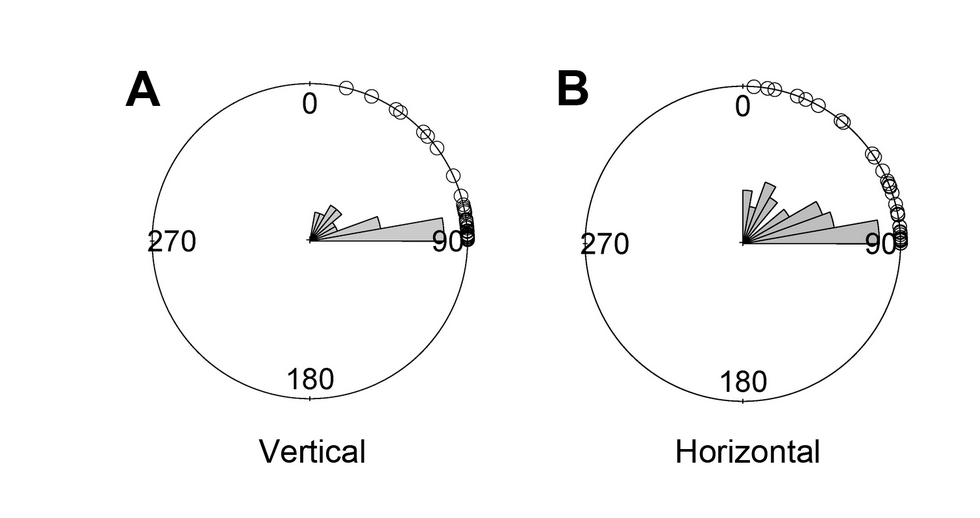

Supplement: Figure S6 — The distribution of head orientations of moths relative to the orientation of the nearest furrow. These figures show the distribution of head orientations of moths on vertical (A), and horizontal (B) log. The angle between the head orientation of a moth and orientation of the closest furrow (closest to the moth’s head) was defined as 0° if moth’s body axis runs parallel to the closet furrow, and to 90° if moth’s head pointed perpendicularly towards the furrow. Rayleigh’s test with specified mean direction (towards 90 °) showed that the moths mostly oriented perpendicularly to the direction of the nearest furrow (vertical treatment: N=35, V=0.91, P<0.001; horizontal treatment: N=34, V=0.82, P<0.001). (TIFF) [file pone.0078117.s006.tiff]
