# Supplementary material for: Cryptically Patterned Moths Perceive Bark Structure When Choosing Body Orientations That Match Wing Color Pattern to the Bark Pattern
Source: PLoS One. 2013 Oct 24;8(10):e78117. doi: 10.1371/journal.pone.0078117 (PMC3813426; doi:10.1371/journal.pone.0078117)
Supplement: Text S1 — Methods and results of the additional tests in experiment 3. (DOCX) [file pone.0078117.s007.docx]

Supporting Information Text for the article:

**Cryptically patterned moths perceive bark structure when choosing body orientations that match wing color pattern to the bark pattern**

Changku Kang*1*, Jong-yeol Moon*1*, Sang-im Lee*1,2*, Piotr G. Jablonski*1,3*

*1* School of Biological Sciences, Seoul National University, Seoul, Republic of Korea

*2*Institute of Advanced Machinery and Design, Seoul National University, Seoul, Republic of Korea

*3*Centre for Ecological Research (previous Institute of Ecology), Polish Academy of Sciences, DziekanowLesny, Poland

Corresponding author:

PiotrJablonski

[**SNULBEE@behecolpiotrsangim.org**](mailto:SNULBEE@behecolpiotrsangim.org)

Supplementary Text S1

*Additional test for evaluating the effect of shadows on moths’ behaviors in the experiment 3*

In the experiment 3 moths did not show preference for orienting towards stronger or weaker shadows. We additionally tested whether moths respond to shadows (or direction of light that comes from) created by directional structures in the vertical treatment of experiment 3. We followed the same procedures as the vertical treatment of the experiment 3, but additionally we put a commercial desk light on the corner of the background to generate shadows only in one side of the directional structure. After the moths settled on the background, we categorized the moths whether they oriented towards the shadowy part of the directional structure or not. This test was done in the warehouse where only dim light were coming through windows.

The number of moths headed towards the shadowy side (10 out of 16) did not significantly differ from the number of moths oriented towards the non-shadowy side (6 out of 16; exact binomial test, *P*=0.45). This suggests that moths’ orientating behaviors were not affected by the shadows created by the directional structural elements of the background.
